# High-resolution fluid-suppressed diffusion tractography of the fornix across the healthy lifespan and deviations in multiple sclerosis

**DOI:** 10.1162/IMAG.a.1186

**Published:** 2026-03-30

**Authors:** Carly Weber, Penelope Smyth, Gregg Blevins, Colin Wilbur, Derek Emery, Christian Beaulieu

**Affiliations:** Biomedical Engineering, University of Alberta, Edmonton, Alberta, Canada; Neurology, University of Alberta, Edmonton, Alberta, Canada; Pediatric Neurology, University of Alberta, Edmonton, Alberta, Canada; Radiology and Diagnostic Imaging, University of Alberta, Edmonton Alberta, Canada

**Keywords:** diffusion tensor imaging, tractography, fornix, multiple sclerosis, lifespan, neurodevelopment, aging

## Abstract

Diffusion tensor imaging (DTI) can infer microstructural changes in white matter tracts like the fornix (main efferent tract of the hippocampus), where there is limited knowledge on typical changes with age from childhood through adulthood, and on whether DTI deviations in multiple sclerosis (MS) are consistent across the lifespan. The objectives are to determine fornix DTI age relationships in healthy controls and characterize DTI deviations in MS. High-resolution, fluid-suppressed DTI tractography quantified fornix volume and DTI parameters (fractional anisotropy - FA; mean, axial, radial diffusivity - MD, AD, RD) in 103 healthy controls (12–65 years) and 42 MS participants (13–63 years). Other brain volumes were measured on T1-weighted MPRAGE. All parameters were assessed for age trajectories, compared between groups, and correlated to clinical/cognitive assessments in MS. Control fornix volume increased markedly (~1.7x) from 12 to 33 years and then declined to a similar extent by 65 years, while MD, AD, and RD showed U-shaped changes with minima ~30–33 years; conversely, FA showed no age relationship. MS fornix volume followed a similar age trend, but remained below controls at all ages and was proportionally reduced (-29%) more than all other brain volumes. In contrast, age relationships of MD, AD, and RD were lost due to similarly higher diffusivity in MS over all ages and FA was below controls across all ages. MS fornix volume/diffusion metrics correlated with various clinical/cognitive scores. Fluid-suppressed, high-resolution diffusion tractography showed nonlinear changes of the fornix in controls over the observed lifespan and early deviations in MS, consistent across age, suggesting it is an early target of injury.

## 1 Introduction

The fornix is the main efferent white matter (WM) tract of the hippocampus and, as part of the limbic system, has been shown to be related to memory and cognition in both healthy individuals over the lifespan as well as affected in many clinical disorders (for reviews, see Douet & Chang (2015) and Benear et al. (2020)). These fornix alterations have most often been assessed with diffusion tensor imaging (DTI), which can provide quantitative metrics (e.g., fractional anisotropy – FA, mean diffusivity – MD) that infer changes in microstructure (related to axon density and/or myelination, for example) and yield 3D virtual reconstructions of the fornix using tractography. To effectively compare disorders, it is first imperative to measure the changes in the fornix with typical “healthy” neurodevelopment and aging over the lifespan.

Infants 1–4 months old have yielded fornix tracts identifiable by DTI tractography that showed FA elevations and MD reductions with age (Dubois et al., 2008). Two DTI studies that have included a wide age span of typical individuals from children/adolescents to elderly have shown non-linear age trends with age showing FA peaks and MD minima ~18–25 years, suggesting that the fornix is an earlier developing white matter tract than others (Lebel et al., 2012; Sala et al., 2012). Other DTI studies have spanned limited age ranges (i.e., children/adolescents to young adults or young adults to elderly), but showed inconsistencies in fornix trends (Douet et al., 2014; Foster et al., 2019; Hu et al., 2025; Jang et al., 2011; Michielse et al., 2010; Sasson et al., 2013; Simmonds et al., 2014; Stadlbauer et al., 2008; Sullivan et al., 2010). Models of diffusion MRI for mid-to-older adult brain age prediction in a large community population study (n = 35,749) identified the fornix as the most contributing brain region with FA decreasing and MD increasing over 45–83 years (Korbmacher et al., 2023). Fornix DTI in typical older adults has shown links with cognitive decline (Fletcher et al., 2013; Peter et al., 2025) and preservation of verbal episodic memory (Ruiz-Rizzo et al., 2024). The importance of detecting DTI changes within the fornix is not just in adults, as typically developing children have shown associations between the fornix and genetic risk factors for schizophrenia (Douet et al., 2014) as well as bipolar disorder and autism spectrum disorder (Fernandez-Cabello et al., 2022). The fornix also appears to have more, earlier age-related changes linked to cognition that precede deleterious alterations of the hippocampus (Foster et al., 2019; Gazes et al., 2019; Metzler-Baddeley et al., 2019).

This predominant contribution of the fornix to memory performance has also been observed in adults with MS (Dineen et al., 2012). Other DTI studies, mostly in adult MS, have shown marked abnormalities in fornix metrics such as reduced volume, lower FA and elevated diffusivities (Valdés Cabrera et al., 2020) and their correlations with cognitive impairments in various measures of memory (i.e., working, visuospatial, episodic, verbal), auditory processing speed, executive functioning, spatial processing, verbal fluency, and fatigue (Keser et al., 2017; Koenig et al., 2013, 2024; Mirmosayyeb et al., 2025; Mistri et al., 2023; Syc et al., 2013; Valdés Cabrera et al., 2022). Recently, DTI tractography has shown similar fornix abnormalities in children/adolescents (e.g., fornix volume smaller by 26%) with pediatric-onset MS (POMS), suggesting that the fornix (but not the hippocampus) is an early brain region affected in MS (Weber et al., 2025). However, there has not been an integrated DTI study covering a wider age range from children to elderly in MS to examine age trajectories of the fornix over the lifespan and how it deviates from that expected in healthy controls.

Such lifespan studies are critical to determine whether brain neurodevelopment and aging changes in MS follow a similar trajectory to that of controls, albeit offset higher/lower, or if they worsen over time. The latter has been reported for brain atrophy in MS over the lifespan (5–73 years) with differences starting ~20 years old (Coupé et al., 2023). A lifespan approach can provide insight into neuropsychology differences from pediatric to adult-onset MS (Nunan-Saah et al., 2015) and in the frequency of cognitive dysfunction in attention/processing speed and episodic memory, which is greater in young adults versus older adults (Vidorreta-Ballesteros et al., 2024). A recent review emphasized that quantitative MRI can be used to investigate the complex links between aging and MS on brain structure and function (Filippi et al., 2024).

The fornix is prone to challenges regarding identification by tractography and diffusion parameter estimation due to its small curvy trajectory and partial volume effects from adjacent rapid, isotropic diffusing cerebrospinal fluid (CSF), given its location in the lateral ventricles (Concha et al., 2005; Vos et al., 2011). None of the prior fornix development/aging studies used CSF suppression in acquisition with fluid attenuated inversion recovery (FLAIR), which can be beneficial for fornix tracking and quantification (Concha et al., 2005). Furthermore, the two prior fornix DTI studies across the healthy “lifespan” (excluding infants and toddlers) had access to older MRI technology (1.5T scanners, limited gradients) and acquired thick slices, that is, 3 mm (Lebel et al., 2012) and 4 mm (Sala et al., 2012). Although our two previous fornix studies on adults with MS (Valdés Cabrera et al., 2022) and children/adolescents with pediatric-onset MS (Weber et al., 2025) used FLAIR-DTI, it has not been used in one integrated study to assess MS changes of the fornix over the “lifespan.”

The purpose here was to use CSF-suppressed, high-resolution FLAIR-DTI tractography to assess fornix changes (i.e., volume and diffusion metrics): (i) versus age across much of the “lifespan” (12–65 years) in healthy controls, (ii) in MS across the same age range, and (iii) their potential correlations with clinical disability and cognitive function.

## 2 Materials and Methods

### 2.1 Participants

This study was approved by the University of Alberta Human Research Ethics Board. All 145 participants provided written informed consent (or parental consent and assent if under 16 years of age), including 42 diagnosed with MS (13–63 years old) that were recruited from the pediatric neuroinflammatory registry and University of Alberta Neurosciences Clinic, and 103 healthy controls (12–65 years old) chosen with a similar age/sex distribution from a previous local normative study using an identical MRI protocol. There were 16 pediatric-onset MS (POMS) diagnosed with MS before 18 years of age and 26 adult-onset MS (AOMS), which together included 35 relapsing-remitting MS (RRMS), 5 secondary progressive MS (SPMS), and 2 primary progressive MS (PPMS). Note that 11/16 of the POMS were included in our prior fornix tractography study (Weber et al., 2025) whereas here, new unpublished AOMS (i.e., no overlap with our former paper (Valdés Cabrera et al., 2022)) have been added to examine changes over the lifespan. The inclusion criteria for the MS cohort was a diagnosis of MS according to the 2017 McDonald criteria. The exclusion criteria included no MS participants with medical diagnoses other than MS or with known clinical relapses at the time of the MRI scan or within 30 days of the study visit. Most (35/42) MS were on disease-modifying therapies (rituximab, ocrelizumab, fingolimod, ofatumumab, dimethyl fumarate, interferon beta-1a, and natalizumab), 19/42 MS had diagnosed depression/anxiety and were on various antidepressant medications, and 5/42 MS had diagnosed attention deficit hyperactivity disorder (ADHD) and were taking ADHD medications. Demographic and clinical data are summarized in Table 1.

**Table 1.** Demographics, cognitive/clinical scores, and total lesion volume for MS and controls with range, mean, and standard deviation.

	Controls (n = 103)	MS (n = 42)
Sex (M/F)^a^	27/76	10/32
Age (years)^a^	12 - 65 33 +/- 15	13 - 63 34 +/- 15
Time since MS onset (years)	-	1 - 31 11 +/- 9
Expanded Disability Status Scale, EDSS^b^	-	0.0 - 8.5 3.0 +/- 2.2
Pediatric fatigue (t-score)	-	47 - 72 60 +/- 8
Modified Fatigue Impact Scale, MFIS (z-score)	-	-0.5 - 5.0 2.4 +/- 1.6
Beck’s Depression Inventory-II, BDI-II^b^	-	1 - 43 15 +/- 11
Symbol Digit Modalities Test, SDMT (z-score)	-	-4.3 - 2.9 -1.0 +/- 2.0
Brief Visuospatial Memory Test-Revised, BVMT-R (total recall) (z-score)^b^	-	-3.5 - 1.4 0.1 +/- 1.3
Timed 25-Foot Walk, T25FW (average of 2 trials, seconds)^b^	-	3.1 - 7.8 4.5 +/- 1.1
Nine-Hole Peg Test, 9HPT (average of 4 trials, seconds)^b^	-	16.3 - 43.6 22.8 +/- 5.6
Total lesion volume, TLV (cm^3^)	-	0.04 - 55.7 12.1 +/- 14.7

a No significant difference in age (t = 0.407, p = 0.685) or sex (χ² = 0.091, p = 0.763).

b EDSS was not acquired from one RR-AOMS. BDI-II was not acquired from one RR-POMS. One SP-AOMS was not able to complete the BVMT-R. T25FW and 9HPT were not acquired from five RR-POMS and 15 AOMS (8 RR, 5 SP, 2 PP).

### 2.2 Cognitive assessment

Cognitive and clinical tests were administered by a trained user and MS neurologists from the University of Alberta, respectively. Tests included: Expanded Disability Status Scale (EDSS) for overall MS disability, Pediatric Fatigue (Neuro-QOL Item Bank v.2.1) for POMS fatigue, Modified Fatigue Impact Scale (MFIS) for AOMS fatigue, Beck’s Depression Inventory-II (BDI-II) for depression, Symbol Digit Modalities Test (SDMT) to measure processing speed, Brief Visuospatial Memory Test-Revised (BVMT-R) to test visuospatial memory (only total recall score), Timed 25-Foot Walk (T25FW) for mobility, and Nine-Hole Peg Test (9HPT) for dexterity. Pediatric Fatigue scores were converted to standardized t-scores based on the 2021 Neuro-QOL scoring Manual (Version 3.0). MFIS (Strober, Bruce, Arnett, Alschuler, DeLuca, et al., 2020), SDMT (Strober, Bruce, Arnett, Alschuler, Lebkuecher, et al., 2020), and BVMT-R (Benedict et al., 1996) scores were converted to standardized z-scores based on normative data. Cognitive/clinical test scores are summarized in Table 1.

### 2.3 MRI protocol

Participants were scanned on a 3T Siemens Prisma MRI with a 64-channel head/neck radiofrequency coil. A whole-brain 3D T1-weighted magnetization-prepared rapid acquisition gradient echo (MPRAGE) was acquired using the following parameters: 0.9 mm isotropic voxels, TR 1800 ms, TE 2.37 ms, and total scan time 3.4 min. Whole-brain 3D sampling perfection with application-optimized contrasts using different flip angle evolution (SPACE) FLAIR was acquired with 1.2 mm isotropic voxels, TI 1800 ms, TR 5000 ms, TE 385 ms, and total scan time 3.1 min. The FLAIR-DTI protocol was the same as used in our previous pediatric and adult MS studies here (Weber et al., 2025; Valdés Cabrera et al., 2022): 2D single-shot echo-planar imaging, 35 2 mm transverse slices centered for fornix coverage, 1.2 × 1.2 mm^2^ zero-filled to 0.64 × 0.64 mm^2^ in-plane resolution, GRAPPA R=2, phase partial Fourier 6/8, TI 2300 ms, TR 9000 ms, TE 70 ms, 5 b = 0 and 20 b = 1000 s/mm^2^, and total scan time 4.1 min.

### 2.4 MRI analysis

MPRAGE brain volumes and SPACE FLAIR total MS lesion volumes were measured using the volBrain and lesionBrain pipelines, respectively (Coupé et al., 2018; Manjón & Coupé, 2016). VolBrain yielded segmented total and regional brain volumes, including: CSF, lateral ventricles, total brain volume (TBV), total white matter (WM), total gray matter (GM), cerebellum (total, WM, GM), caudate, putamen, globus pallidus, thalamus, hippocampus, and amygdala. Left and right hemispheres were combined to limit comparisons and yielded 14 volumes per participant. LesionBrain yielded total lesion volume (TLV).

DTI processing was performed in MRtrix3 (v.2.0), including denoising, Gibbs ringing, eddy current and motion, bias field correction, and tensor fitting (Tournier et al., 2019). Deterministic tractography of the fornix was performed in MRtrix3 similar to previously published lab protocols (Valdés Cabrera et al., 2022; Weber et al., 2025) using: FA threshold 0.15, maximum turning angle 65^o^, step size 0.64 mm, minimum fiber length 10 mm, and maximum fiber length of 109 mm (i.e., MRtrix3 default, geometric mean of interpolated voxel size times 100). Regions of interest (ROIs) were placed as follows: coronal AND ROI in the fornix body, axial NOT ROI to remove tracts superior to the fornix, coronal NOT ROI to remove tracts anterior to the fornix columns, coronal NOT ROI to remove tracts posterior to the fornix crura, and other NOT ROIs as needed to remove all remaining streamlines not attributable to the fornix. This tract yielded the whole fornix volume, as well as average FA, MD, AD, and RD across left and right sides. A binary mask was generated from the tractography, whereby each voxel contributed once to the average, independent of the number of streamlines passing through each voxel.

### 2.5 Statistical analysis and curve fitting

Fornix volume, FA, MD, AD, and RD, and total/regional brain volumes for MS and controls were each fit versus age to linear, Poisson, quadratic, or cubic models based on best fit, which was determined by comparing the Akaike Information Criterion (AIC) values of each model and selecting the model with the lowest AIC value (Virtanen et al., 2020). If AIC values were within two units of each other, models were considered equally fit and the simplest model was chosen (i.e., the model with the fewest parameters) (Burnham & Anderson, 2004). Only significant fits (p < 0.05) are shown.

Fornix volume, FA, MD, AD, and RD, and total/regional brain volumes for MS and controls were tested for normality, and then metrics were assessed with Mann-Whitney U tests to test for group differences. Pearson correlations were used to assess linear relationships between fornix volume/diffusion metrics (and other total/regional brain volumes) and clinical/cognitive scores, that is, time since MS onset, EDSS, TLV, Pediatric Fatigue, MFIS, BDI-II, SDMT, BVMT-R, T25FW, and 9HPT. False discovery rate (FDR) corrected p-values are presented (*p < 0.05).

## 3 Results

**Fig. 1.**
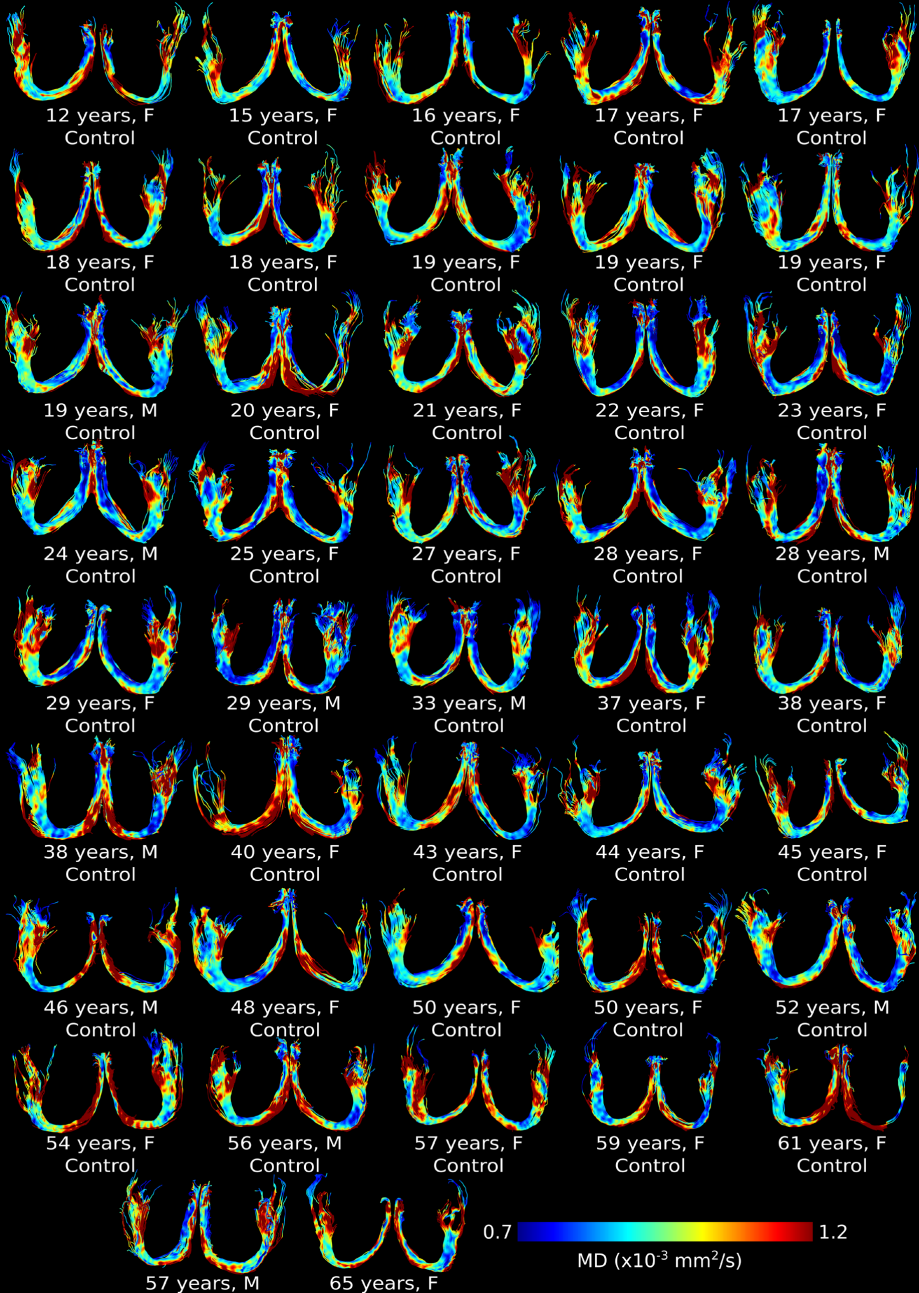
Example of CSF-suppressed, high-resolution FLAIR-DTI fornix tractography (as seen from superior view) color-coded by MD in 42 representative age/sex matched controls, ordered by age. Fornix tractography depicted the full fornix in all and showed bilateral regions with higher MD after the fourth decade of life.

**Fig. 2.**
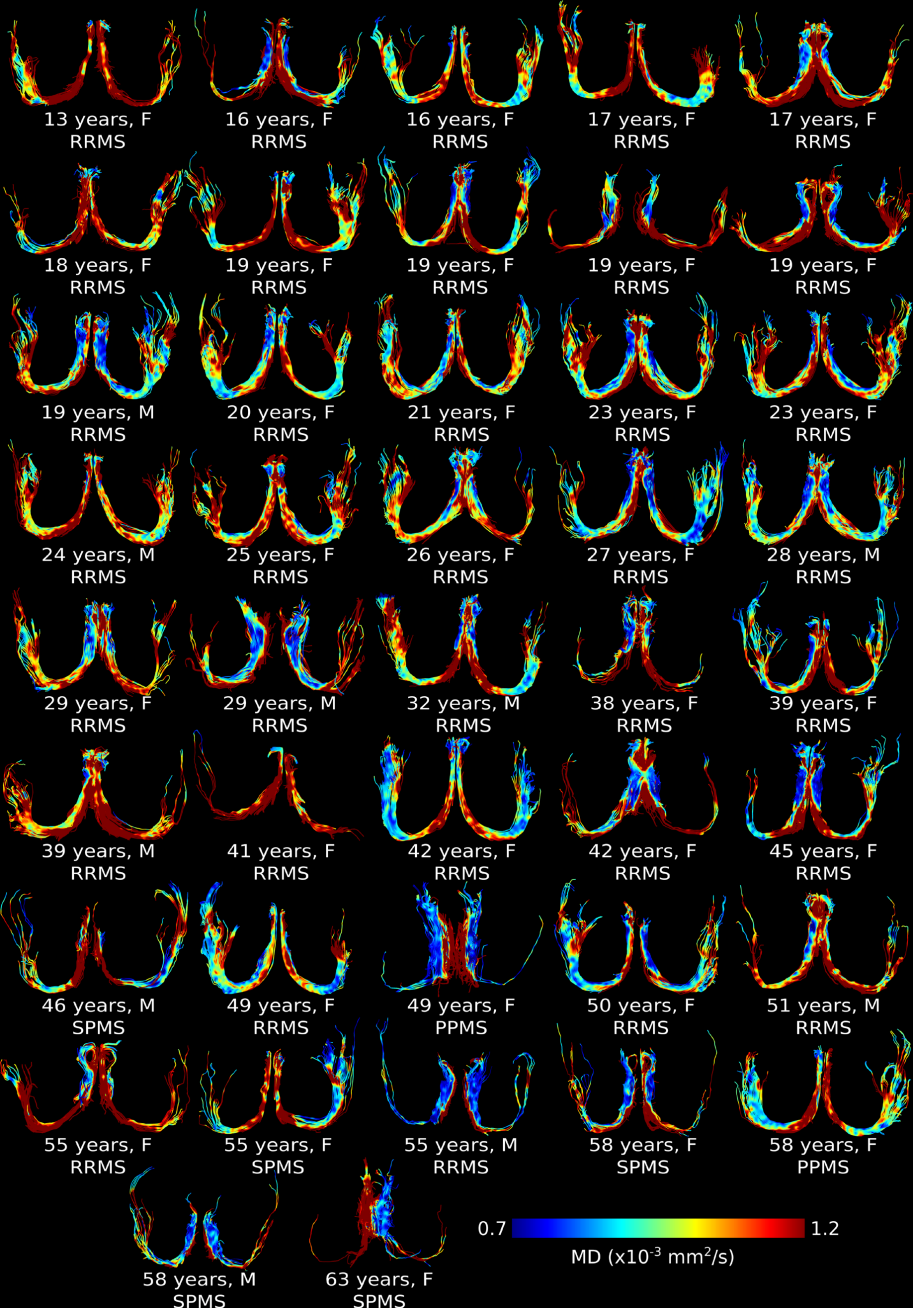
Example of CSF-suppressed, high-resolution FLAIR-DTI fornix tractography (as seen from superior view) color-coded by MD in all 42 MS, ordered by age. Fornix tractography depicted the full fornix in all, but it appeared thinner and showed bilateral regions with higher MD at most ages.

CSF-suppressed, high-resolution FLAIR-DTI tractography depicted the full fornix in all 103 controls and 42 MS participants (see Fig. 1 for 42 representative age/sex matched control fornix and Figure 2 for all 42 MS fornix, colour-coded by MD). Fornix tractography showed bilateral regions with higher MD after the fourth decade of life in controls and thinner fornix (especially progressive MS) with higher MD across most ages in MS compared to controls. Mean DWI b1000, FA and MD maps also showed differences between controls and MS across most ages (Supplementary Fig. S1).

**Fig. 3.**
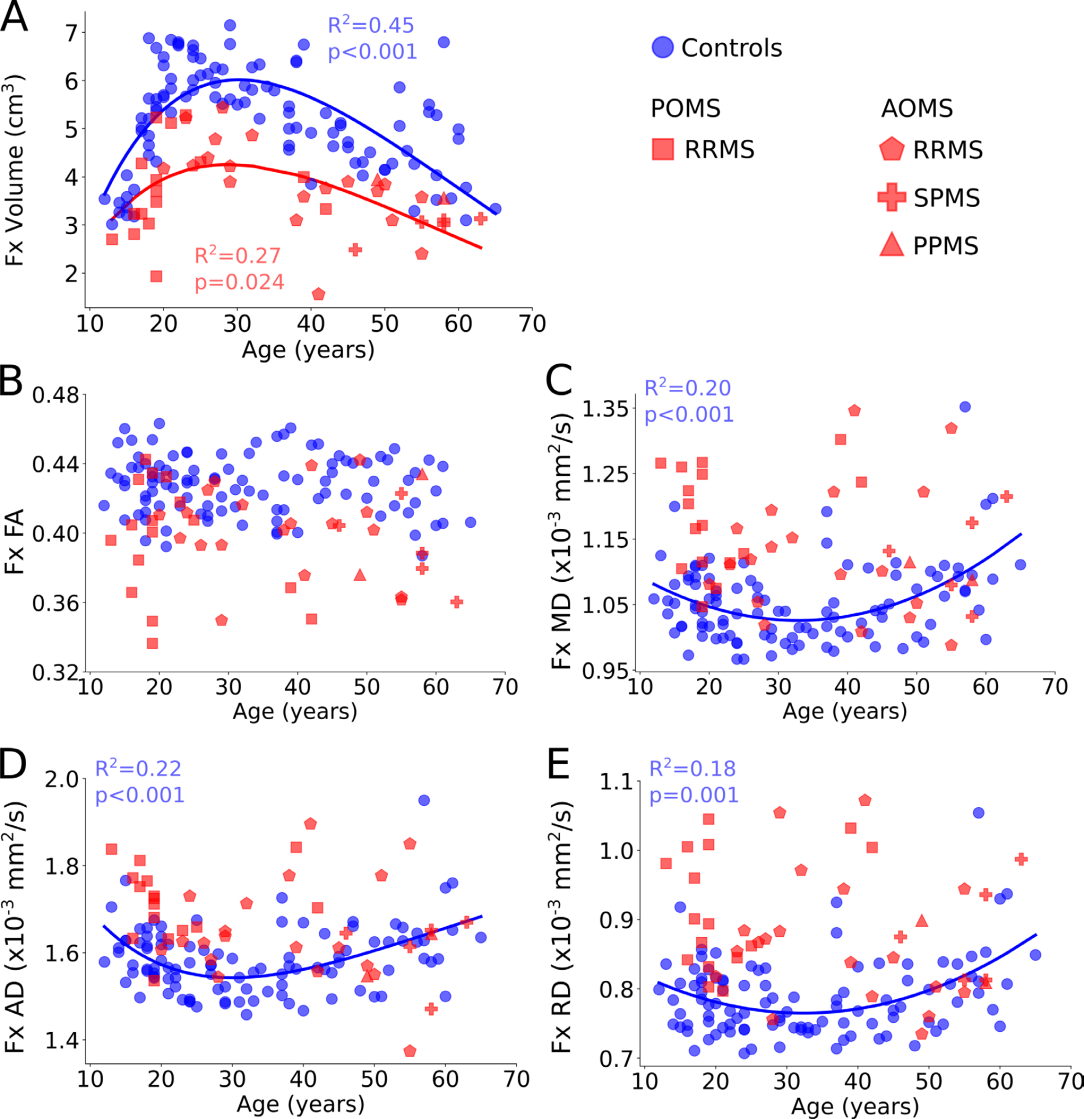
Fornix (Fx) (A) volume, (B) FA, (C) MD, (D) AD and (E) RD shown vs. age for controls (n = 103, in blue) and pediatric-(PO) and adult-onset (AO) MS (n = 42, in red). Fornix volume in MS followed a Poisson curve similar to controls, but peaked earlier (29 vs. 33 years) and remained below controls across all ages. Control fornix FA showed no age relationships, whereas MD, AD, and RD showed similar curves with age (minimums at 33, 30, and 32 years, respectively), although there were no age relationships for MS fornix diffusion metrics, which remained mainly below (i.e., FA) and above (i.e., MD, AD, and RD) controls at all ages.

Poisson curves fit control and MS fornix volume versus age (R^2^ = 0.45, p < 0.001 and R^2^ = 0.27, p = 0.024, respectively) (Fig. 3A), both of which became larger from 12 and 13 years until peaking at 33 and 29 years of age with increases of ~70% and ~56%, respectively, and then became smaller with age toward 63–65 years at a slower rate than the upswing ending up with volumes similar to that at 12 and 13 years of age. Notably, the fornix volume of the MS group remained below controls across all ages. Control and MS fornix FA did not show any significant age relationships, but ~1/3 of the MS participants had FA below all controls across the full age span and the majority were below the mean of the controls (Fig. 3B). Control fornix MD (quadratic, R^2^ = 0.20, p < 0.001, Fig. 3C), AD (Poisson, R^2^ = 0.22, p < 0.001, Fig. 3D). and RD (quadratic, R^2^ = 0.18, p = 0.001, Fig. 3E) followed similar trends with age where they decreased until 33, 30, and 32 years of age, respectively, and then increased to a greater extent with aging. In contrast, fornix MD, AD, and RD in MS participants did not show significant age relationships, but most were larger than the control mean across most ages, including MD, AD, and RD for under 20 years (+12%; +8%; +16%), 20–29 years (8%; 5%; 10%), and 30–39 years (16%; 12%; 19%) (p < 0.001), as well as MD and RD for 40–49 years (9% and 13%) (p < 0.05), but were not significantly different after 50 years.

**Fig. 4.**
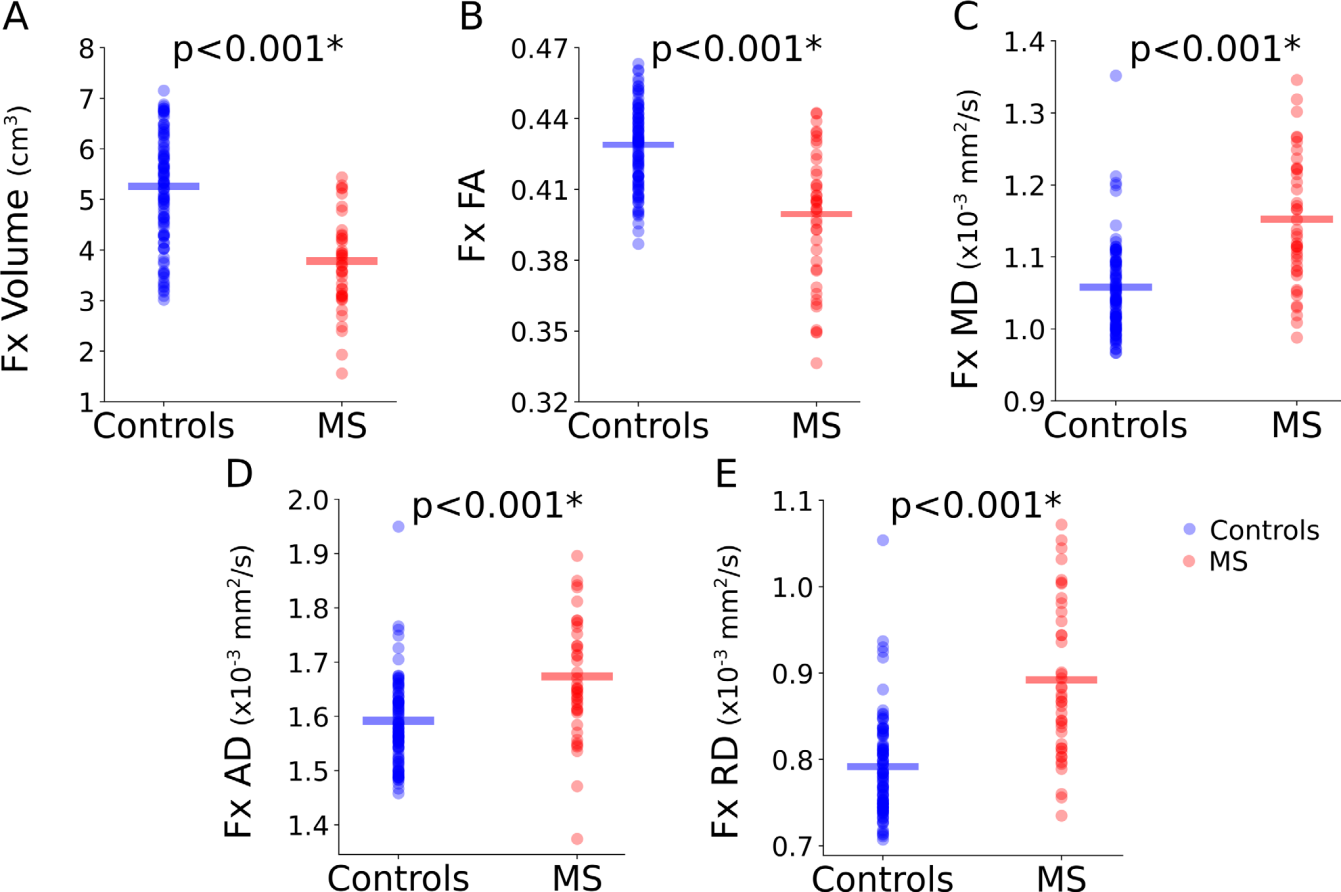
Group comparisons of controls (n = 103) and MS (n = 42) are shown for fornix (Fx) (A) volume, (B) FA, (C) MD, (D) AD, and (E) RD. MS fornix showed a smaller volume (-29%) compared to controls as the largest change in the fornix, in addition to significant differences in FA (7% lower), MD (10% higher), AD (6% higher), and RD (13% higher).

Averaged over all ages relative to controls, MS fornix were 29% smaller (5.2 +/- 1.1 vs. 3.7 +/- 0.9 cm^3^, p < 0.001), with 7% lower FA (0.43 +/- 0.02 vs. 0.40 +/- 0.03, p < 0.001), 10% higher MD (1.05 +/- 0.06 vs. 1.15 +/- 0.09 × 10^-3^ mm^2^/s, p < 0.001), 6% higher AD (1.58 +/- 0.08 vs. 1.67 +/- 0.11 × 10^-3^ mm^2^/s, p < 0.001), and 13% higher RD (0.79 +/- 0.06 vs. 0.89 +/- 0.09 × 10^-3^ mm^2^/s, p < 0.001) (Fig. 4). Considering all regional volumes (Table 2), MS had the greatest proportional volume differences compared to controls in the lateral ventricles (+81%) and CSF (+28%), followed by thalamus (-21%), cerebellum WM (-18%), total WM (-14%), globus pallidus (-13%), putamen (-12%), caudate (-9%), TBV (-8%), total cerebellum (-7%), amygdala (-6%), and hippocampus (-5%). Note that hippocampus volume (age relationships reported below) was not different between POMS (under 20 years) and MS > 20 years old (7.8 +/- 0.7 vs. 7.6 +/- 1.1 cm^3^, p = 0.662) or controls vs. MS under 35 years (8.1 +/- 0.7 vs. 7.9 +/- 0.7 cm^3^, p = 0.474), but MS showed smaller hippocampus (-11%) vs. controls for 35 years and above (8.3 +/- 0.9 vs. 7.3 +/- 1.2 cm^3^, p = 0.002). There were no differences in total GM or cerebellum GM volumes, and the above regional brain volume proportional reductions were not as great as the fornix (-29%).

**Table 2.** Total/regional left + right WM/GM volumes (cm^3^) and fornix volume (cm^3^) with group comparisons for age/sex matched controls versus MS (mean +/- SD), ordered from greatest positive percentage changes and then greatest negative percentage changes (FDR corrected; *p < 0.05; n.s. is non-significant).

Volumes (cm^3^)	Controls (n = 103)	MS (n = 42)	Difference (%)	
Lateral ventricles	11.3 +/- 6.5	20.4 +/- 14.7	+81	p < 0.001*
CSF	174 +/- 48	223 +/- 75	+28	p = 0.001*
Fornix	5.2 +/- 1.1	3.7 +/- 0.9	-29	p < 0.001*
Thalamus	12.6 +/- 1.2	10.0 +/- 2.0	-21	p < 0.001*
Cerebellum WM	38 +/- 7	31 +/- 8	-18	p < 0.001*
WM (total)	546 +/- 58	471 +/- 84	-14	p < 0.001*
Globus pallidus	2.4 +/- 0.3	2.1 +/- 0.4	-13	p = 0.001*
Putamen	8.9 +/- 0.9	7.8 +/- 1.3	-12	p < 0.001*
Caudate	7.6 +/- 1.0	6.9 +/- 1.1	-9	p = 0.001*
TBV	1281 +/- 111	1183 +/- 137	-8	p < 0.001*
Cerebellum (total)	144 +/- 14	134 +/- 20	-7	p = 0.016*
Amygdala	1.7 +/- 0.2	1.6 +/- 0.2	-6	p = 0.004*
Hippocampus	8.1 +/- 0.8	7.7 +/- 1.0	-5	p = 0.019*
GM (total)	735 +/- 76	713 +/- 73	n.s.	p = 0.149
Cerebellum GM	106 +/- 13	103 +/- 15	n.s.	p = 0.542

There were no sex differences between MS males vs. females in fornix volume, FA, MD, AD, or RD (or control males vs. females, except for fornix volume), although fornix volume/diffusion metrics were significantly different between control and MS males and females, separately (Supplementary Table S1). Importantly, the same fornix volume/diffusion changes were shown for controls vs. MS in males and females separately: all participants MS fornix volume reduction -29% vs. males/females separately -31%/-28%, FA -7% vs. -9%/-7%, MD +10% vs. +8%/+10%, AD +6% vs. +3%/+7%, and RD +13% vs. +12%/+13%.

**Fig. 5.**
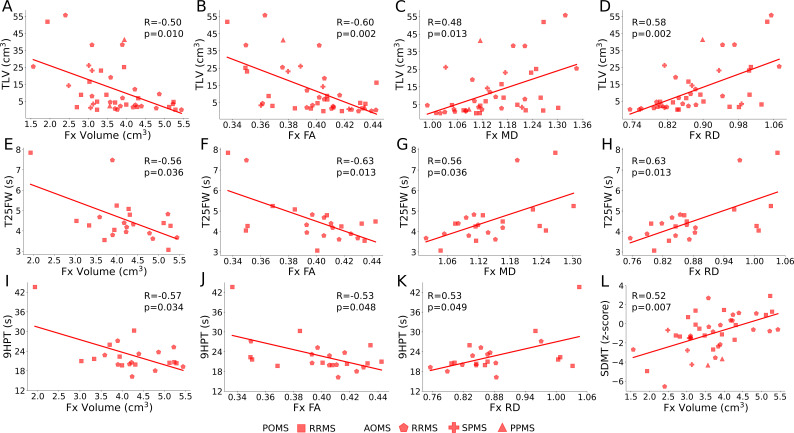
In MS, larger total lesion volume (TLV) (A-D) and worse (i.e. slower) timed 25 foot walk (T25FW) (E-H) and 9-hole peg test (9HPT) (I-K) correlated with smaller fornix volume and FA, and larger fornix MD (excluding 9HPT) and RD. Better symbol digit modality test (SDMT) scores (i.e., higher scores) correlated with larger fornix volume (L).

For MS fornix, significant Pearson correlations (FDR-corrected) were found for TLV vs. fornix volume (R = -0.50, p = 0.010), FA (R = -0.60, p = 0.002), MD (R = 0.48, p = 0.013) and RD (R = 0.58, p = 0.002) (Fig. 5A-D), SDMT vs. fornix volume (R = 0.52, p = 0.007) (Fig. 5L), T25FW vs. fornix volume (R = -0.56, p = 0.036), FA (R = -0.63, p = 0.013), MD (R = 0.56, p = 0.036), and RD (R = 0.63, p = 0.013) (Fig. 5E-H), and 9HPT vs. fornix volume (R = -0.57, p = 0.034), FA (R = -0.53, p = 0.048), and RD (R = 0.53, p = 0.049) (Fig. 5I-K). Interestingly, fornix volume only correlated with hippocampus volume in controls (not MS) (R = 0.27, p = 0.030) and fornix diffusion metrics did not correlate with hippocampus volume in controls or MS (data not shown). No significant correlations were found between fornix volume/diffusion metrics and time since MS onset, EDSS, Pediatric Fatigue, MFIS, BDI-II or BVMT-R; however, other global/regional structures (i.e., CSF, lateral ventricles, TBV, total WM, total GM, cerebellum total/WM/GM, caudate, putamen, globus pallidus, hippocampus, and amygdala) correlated with these measures as well as TLV, SDMT, T25FW, and 9HPT (Supplementary Table S2).

MPRAGE T1-weighted brain volumes from volBrain also showed age correlations, including global (Supplementary Fig. S2) and deep GM volumes (Supplementary Fig. S3).

## 4 Discussion

### 4.1 Fornix DTI tractography in normative controls over “Lifespan”

Quantitative study of the fornix, a critical limbic system tract, often with DTI tractography, can be greatly improved using a protocol designed for the fornix with FLAIR-DTI acquisition to null CSF signal, minimizing deleterious partial volume effects (and limiting slice coverage to just the fornix, yielding a short scan time of ~4 min at 3T), and higher resolution (~3x smaller voxel volumes) than typically used for whole-brain DTI given its small size (Concha et al., 2005). This methodology was applied for the first time here to study development and aging trajectories of the fornix over a significant portion of the typical healthy lifespan covering 12 to 65 years (to match the age range of the MS participants). There were marked non-linear age trajectories such as an asymmetric inverted U-shaped Poisson curve for volume and a U-shaped quadratic curve for mean diffusivity (and AD and RD) that generally agreed with two earlier studies at 1.5T with no CSF suppression and thick slices, which included a wide age range of healthy controls (5–83 years in Lebel et al., 2012, 13–70 years in Sala et al., 2012). The MD drops ~5% from 12 years to the minima and then goes up by ~12% to 65 years—these proportional changes are in excellent agreement with (Lebel et al., 2012). The peak/minima in the early 30s is older than the 18–20 years reported in Lebel et al. (2012) that suggested it was an early developing tract, although the DTI-derived values are quite similar across 20–30 years in their curve fits, as well as ours. However, peak/minima ~30 years fits with most of the other white matter tracts reported in that study. In contrast, Sala et al. (2012) show similar MD values of the fornix from 12–30 years, only increasing exponentially afterwards.

Notably, hippocampus volume does not change with age in our normative cohort, whereas fornix volume shows marked age-related change. Fornix volume nearly doubles (increasing ~70% from ~3.5 cm^3^ to ~6 cm^3^) over two decades, followed by a more gradual decline over the next 3.5 decades back to its original size by 12 years (decreasing 40% from ~6 cm^3^ to ~3.5 cm^3^). Other total/regional and deep GM brain volume trends (i.e., positive relationships for CSF and lateral ventricles, and negative relationships for total GM, caudate, putamen, globus pallidus, and thalamus) are consistent with previous work (Bartels et al., 2019; Eshaghi et al., 2018; Good et al., 2001; Lebel et al., 2012; Michielse et al., 2010; Narvacan et al., 2017; Treit et al., 2022). Even the flat trajectory shown for control hippocampus volume with age in this study agrees with an independent measure of hippocampus volume (manual segmentation of 1 mm DWI) in the same cohort of controls if the age range was restricted to the same age range used here, regardless of differences in hippocampus volume with manual segmentation (Solar et al., 2021). However, age relationships (for hippocampus and in general) may be largely driven by controls above 65 years (missing in this study). Nonetheless, the greater changes observed in the fornix here are consistent with reports suggesting it has stronger associations with age-related decline and cognition than the hippocampus (Gazes et al., 2019; Metzler-Baddeley et al., 2019). In terms of histopathology, light and electron microscopy of the primate fornix across the macaque lifespan showed no change in the cross-sectional area of the fornix using a linear fit (only linear fits were assessed), but Figure 3A in Peters et al. (2010) suggests an increase from 4 years (young) to 15 years (middle aged), followed by a reduction toward 33 years (elderly), similar to the fornix volume trajectory here. The elevated MD shown here with age also fits with their linear reductions of total number of myelinated fibers and mean density of fibers with age in the macaques, and there appears to be an upswing from young to middle age (see their Fig. 3B), which would fit with an observed reduction of MD in the fornix during development (Peters et al., 2010). Genetic mice models have also shown a link between oligodendrogenesis and fornix volume during the juvenile period, which fits with the fornix volume trajectory here (Gazdzinski et al., 2025).

The impact of the FLAIR-DTI protocol here is evident when comparing the parameters reported in the two prior lifespan fornix DTI papers. As a result of fuller tracking without streamline discontinuities, our tractography derived peak fornix volume is nearly double that of Lebel et al. (2012) (6 cm^3^ vs. 3.3 cm^3^). The MD values are lower with FLAIR-DTI protocol yielding best fit age ranges of 1.03–1.16 × 10^-3^ mm^2^/s vs. 1.15–1.30 × 10^-3^ mm^2^/s (Lebel et al., 2012) vs. 1.18–1.60 × 10^-3^ mm^2^/s (Sala et al., 2012). Fornix FA in our study showed no changes across age reflecting similar changes in AD and RD unlike Lebel et al. (2012), although they showed a very shallow Poisson trajectory with FA changes less than 0.01 over 12–65 years in their best fit and Sala et al. (2012) showed larger FA drops (by 0.10) after 40 years. Absolute FA values were quite similar across the 3 studies at ~0.41–0.43 for younger ages, which suggests the FA changes with older age seen in their work may be the result of partial volume, particularly in Sala et al. (2012), which used an atlas-based region-of-interest approach (Sala et al., 2012). However, this finding could also be due to the inclusion of younger ages in Lebel et al. (2012) and older ages in Lebel et al. (2012) and Sala et al. (2012) that drive age relationships. Other fornix DTI studies in healthy adult aging (i.e., no children) including elderly individuals (i.e., up to 94 years in Foster et al., 2019 and 80 years in Korbmacher et al., 2023) have shown large FA reductions, but this may be partly attributed to partial volume with isotropic, rapidly diffusing CSF given the lower spatial resolution without CSF suppression protocols (Korbacher et al., 2023; Foster et al., 2019). Differing partial volume effects likely confound fornix diffusion changes as the proportion of CSF contributing to a voxel increases when there is atrophy (Vos et al., 2011), such as in MS.

### 4.2 Fornix DTI tractography in multiple sclerosis over “Lifespan”

Relative to controls, the fornix over all MS participants combined were smaller by 29%, which makes it proportionally more affected than any other WM or GM region volume loss assessed in this study (range of -5% for hippocampus to -21% for thalamus, in agreement with previous literature, see Aubert-Broche et al., 2011; Bartels et al., 2019; De Meo et al., 2019; Eshaghi et al., 2018), and had lower FA (7%) and greater diffusivities (6% AD, 10% MD, 13% RD). These fornix volume/diffusion metrics were changed to a similar extent as in our previous cross-sectional adult-only MS study (with no overlapping patient scans with current paper), which used the same FLAIR-DTI tractography protocol (Valdés Cabrera et al., 2022): this lifespan study MS volume -29% vs. prior adult MS study -26%, FA -7% vs. -7%, MD +10% vs. +9%, AD +6% vs. +6%, and RD +13% vs. +12%. Importantly, the above results were similar regardless of the different DTI post-processing software used in either study. Note that fornix volume is reduced by 28% if total intracranial volume (reduced in MS by 3%) is corrected per participant and by 24% if total brain volume (reduced in MS by 8%) is corrected per participant (data not shown). This marked reduction of fornix volume is similar to other neurological disorders linked with dementia in the elderly such as cerebral amyloid angiopathy, mild cognitive impairment and Alzheimer’s Disease, which all also showed ~30% volume reductions compared to controls using FLAIR-DTI tractography (Shaikh et al., 2022). It is important to emphasize that the fornix findings observed here were also shown in children and adolescents with MS (Weber et al., 2025).

The age trajectory showed that MS fornix volume followed a similar inverted asymmetric U-shaped Poisson curve as controls, but was below controls across all ages with minimal overlap. The fornix in MS still showed an upswing during development, albeit with a smaller ~56% increase from 13 years to the peak (vs. 70% in controls), peaked a bit earlier at 29 years (vs. 33 years in controls), and then showed a downswing with aging with a smaller ~26% decrease from the peak to 63 years (vs. 40% in controls), providing evidence that fornix volume is affected early on in the MS disease course. This also suggests that fornix injury is not progressive in MS, although testing this would require longitudinal scans. There were no obvious differences across MS subtypes of RRMS, SPMS, and PPMS, in agreement with earlier studies (Valdés Cabrera et al., 2020). Notably, the decline in fornix volume with age after 29 years old is consistent with the negative relationship shown for fornix volume in adult MS spanning young adults to elderly (Valdés Cabrera et al., 2022). Fornix volumes in MS stayed consistently lower and do not diverge from controls with age; however, other brain volumes do change more with age in MS such as greater CSF and lateral ventricles and lower total brain volume, total GM, cerebellum, cerebellum GM, putamen, and hippocampus, which is in agreement with previous studies (thalamus and globus pallidus volumes did not change with age, likely due to marked volume reductions at all ages, similar to the fornix) (Bartels et al., 2019; Eshaghi et al., 2018). The hippocampus in MS was only smaller compared to controls after 35 years supporting the idea that fornix atrophy occurs earlier, as suggested in typical aging studies (Gazes et al., 2019; Metzler-Baddeley et al., 2019). Similar to the previous adult MS study, hippocampus volume did not correlate with fornix volume in MS, unlike controls (Valdés Cabrera et al., 2022). This suggests that fornix microstructure and volume loss is affected early on in the disease course of MS before the hippocampus by demyelination and/or axonal loss from adjacent CSF factors, not Wallerian degeneration from neuronal death in the hippocampus (Bodini et al., 2016; Pardini et al., 2021). Some fornix volume loss may be attributed to the loss of axons projecting from the subiculum to the anterior thalamic nuclei, which passes along the anterior portion of the fornix, but this study would require higher spatial resolution acquisition (Senova et al., 2020).

Like controls, the fornix in MS did not show changes of FA with age, similar to our previous adult MS study on an independent cohort (Valdés Cabrera et al., 2022), but the majority of MS participant FA values were below the mean FA of controls across all ages. Unlike controls, which showed U-shaped relationships for the diffusivities versus age, MS fornix diffusivities (MD, AD, RD) did not show age relationships, but most were above controls across all ages, especially fornix RD. The pathological effects on the fornix likely overwhelmed the expected age relationships of FA and diffusivities shown in controls. The fornix DTI changes were even similar between POMS (13–19 years) and progressive MS (46–63 years), providing further evidence for early fornix injury in MS. The largest percent change in diffusion metrics of fornix RD, over AD, suggests that injury may be largely from demyelination in the fornix (Concha et al., 2010; Klawiter et al., 2011; Song et al., 2002). However, axonal loss (i.e., lower density), which may be specific to AD, is also possible and would explain the markedly smaller fornix volume shown for MS compared to controls (Concha et al., 2006; Klistorner et al., 2018; Song et al., 2002, 2003). This early injury of the fornix (located within the ventricles, largely surrounded by CSF) would fit with a “surface-in pathology” hypothesis for MS, suggesting exposure to soluble inflammatory factors in CSF (Pardini et al., 2021).

### 4.3 Clinical and demographic correlations with fornix DTI and other regional volumes

MS fornix volume and diffusion metrics had significant linear correlations with several characteristics, such as total lesion volume (TLV) and T25FW (both positive for MD and RD, negative for volume and FA), SDMT (positive for volume), and 9HPT (negative for volume and FA, positive for RD) (Fig. 5, Supplementary Table S2). The TLV correlations agree with our prior adult and POMS studies using the same protocol (Valdés Cabrera et al., 2022; Weber et al., 2025); note that TLV correlates with nearly every measure (15/19). However, the positive correlation found for SDMT vs. fornix volume was not significant in the previous studies, which may be the result of the larger sample size used here. SDMT also correlated significantly with fornix FA, MD, and RD in the previous studies (Koenig et al., 2024; Mirmosayyeb et al., 2025; Valdés Cabrera et al., 2022; Weber et al., 2025), but not in this study, which may reflect the nonlinear changes shown for fornix microstructure with age here. Importantly, other studies have linked the T25FW and 9HPT to other WM bundles in MS (Cordani et al., 2022; Klineova et al., 2016; Sbardella et al., 2013), but only one study has shown positive/negative correlations for the 9HPT vs. fornix FA and MD, respectively (Syc et al., 2013). It is important to note that T25FW and 9HPT scores were only acquired in 22/42 MS participants. Notably, fornix metrics did not correlate with time since MS onset, EDSS, or memory (BVMT-R), similar to previous fornix DTI studies in adult MS (Koenig et al., 2013; Valdés Cabrera et al., 2022), excluding Syc et al. (2013); however, many brain volumes did correlate with these. Finally, fatigue (MFIS) and depression (BDI-II) showed no correlations to any brain region.

### 4.4 Limitations, other considerations, and conclusion

There were several limitations in the current study, including the limited age range of controls and MS participants, which were missing very young (<12 years) and older (>65 years) individuals. However, this study analyzed all sex-matched controls from a previous normative study within the same age range as the MS participants that had identical FLAIR-DTI. There were also few male MS subjects, limiting the analysis of sex effects. Fornix volume/diffusion metrics also appeared worse in two POMS subjects who were scanned as adults (39 and 42 years old) than other AOMS in the same age range, however future work is needed to determine whether these findings persist in larger sample sizes. The focus on a single tract of interest was another limitation as it prevented the ability to conclude whether findings were limited to the fornix or extended to other WM tracts. FLAIR-DTI has limited brain coverage as it was developed for the fornix (70 mm coverage by 35 slices 2 mm thick), but future analyses will include whole-brain multi-shell diffusion MRI analysis of other WM tracts. Due to its inversion pulse and long inversion time to null long T1 CSF, the addition of the FLAIR pulse to DTI extends the acquisition time; it also lowers signal-to-noise ratio. Most studies do not prospectively acquire such CSF-nulled diffusion MRI data for the fornix, but rather retrospectively analyze whole-brain, lower-resolution, diffusion MRI data. In such cases, post-processing methods are the only option, such as free water elimination, which uses a two pool model to estimate “free water” that has been applied to MS showing a higher free water fraction (16–20%) in the fornix, although without any diffusion parameter differences, in disagreement with our FLAIR-DTI findings (Beaudoin et al., 2021). Their study used 2 mm isotropic resolution, which is not ideal for small tracts, but they did acquire two non-zero b value shells (300 and 1000 s/mm^2^) as more than one is required for free water estimation (Hoy et al., 2015), although this is not often the case for most studies.

The findings presented in this study provide valuable insights about fornix macro- and micro-structure changes across the “lifespan” in MS, which deviate consistently from nonlinear age changes observed in controls (which are consistent with previous normative lifespan studies, i.e., inverted U-shaped Poisson curve for fornix volume and U-shaped curves for fornix diffusivities). These findings suggest that fornix micro-structure in MS may be affected early rather than undergoing late, progressive degradation, possibly due to its close proximity to CSF, and that this injury is associated with decline in clinical disability/cognitive function. Given its role as an important WM tract implicated in a number of neurological disorders, the fornix, when assessed using appropriate DTI methodology (i.e., CSF-suppressed, high resolution), may serve as a sensitive marker for detecting and understanding a variety of disease processes, such as those in MS.

## Supplementary Material

Supplementary Figure S1

Supplementary Figure S2

Supplementary Figure S3

Supplementary Table S1

Supplementary Table S2

## Data Availability

For data and code access, please email the principal investigator, Christian Beaulieu at christian.beaulieu@ualberta.ca.
