# Supplementary material for: High-resolution fluid-suppressed diffusion tractography of the fornix across the healthy lifespan and deviations in multiple sclerosis
Source: Imaging Neurosci (Camb). 2026 Mar 30;4:IMAG.a.1186. doi: 10.1162/IMAG.a.1186 (PMC13037659; doi:10.1162/IMAG.a.1186)
Supplement: Supplementary Figure S1 [file IMAG.a.1186_Figure_S1.pdf]

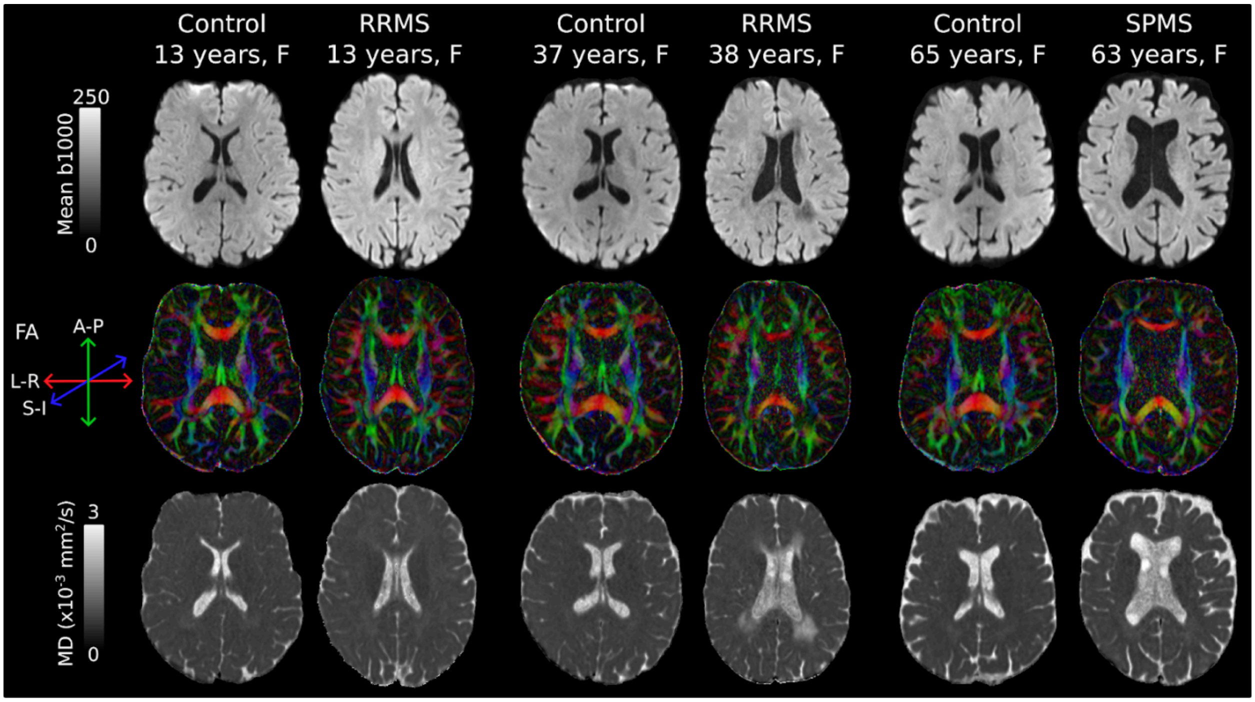

Supplemental Figure S1: DTI maps (mean DWI b1000, top row; colour FA, middle row; and MD, bottom row) for controls and MS of various ages across the lifespan. Lateral ventricle volume appears to get larger with age, particularly in MS, in addition to greater lesion volumes. The fornix (central green inverted v) can be seen on the colour FA map.
