# Supplementary material for: High-resolution fluid-suppressed diffusion tractography of the fornix across the healthy lifespan and deviations in multiple sclerosis
Source: Imaging Neurosci (Camb). 2026 Mar 30;4:IMAG.a.1186. doi: 10.1162/IMAG.a.1186 (PMC13037659; doi:10.1162/IMAG.a.1186)
Supplement: Supplementary Figure S2 [file IMAG.a.1186_Figure_S2.pdf]

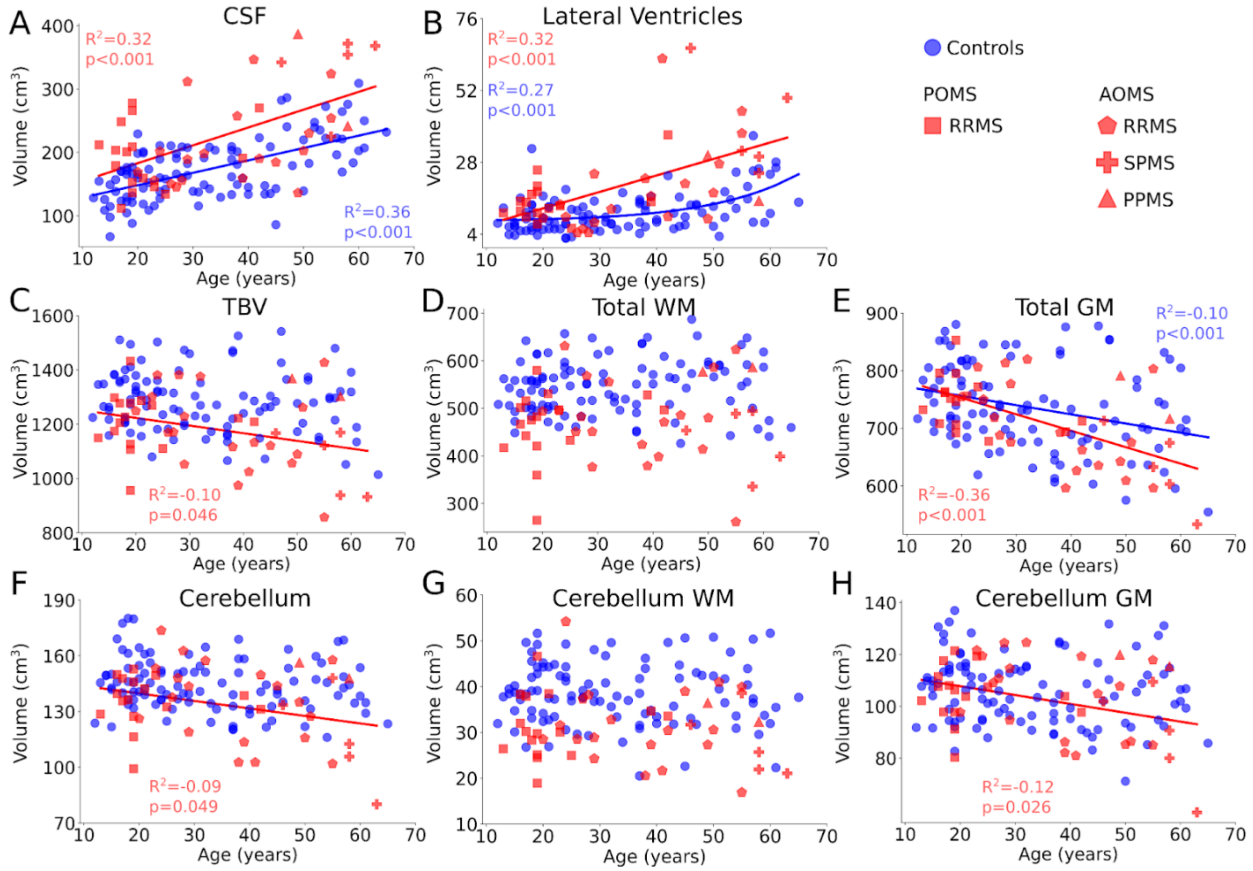

Supplemental Figure S2: Global volumes (left+right) of (A) CSF, (B) lateral ventricles, (C) TBV, (D) total WM, (E) total GM, (F) cerebellum (total), (G) cerebellum WM and (H) cerebellum GM versus age for controls (n=103. blue) and pediatric- and adult-onset MS (n=42, red). Control and MS CSF and lateral ventricle volumes showed positive relationships with age, with steeper slopes for MS over all ages. The control and MS total GM showed negative relationships with age, with a steeper slope for MS. MS TBV, cerebellum, and cerebellum GM showed negative age relationships. Notably, total GM and cerebellum GM were not significantly different in MS as a group (Table 2). Neither total WM or cerebellum WM showed age relationships in either group, although WM volumes were smaller in a significant proportion of MS across all ages (~1/3 of the MS participants had smaller volumes than controls spanning the full age range).
