# Supplementary material for: High-resolution fluid-suppressed diffusion tractography of the fornix across the healthy lifespan and deviations in multiple sclerosis
Source: Imaging Neurosci (Camb). 2026 Mar 30;4:IMAG.a.1186. doi: 10.1162/IMAG.a.1186 (PMC13037659; doi:10.1162/IMAG.a.1186)
Supplement: Supplementary Figure S3 [file IMAG.a.1186_Figure_S3.pdf]

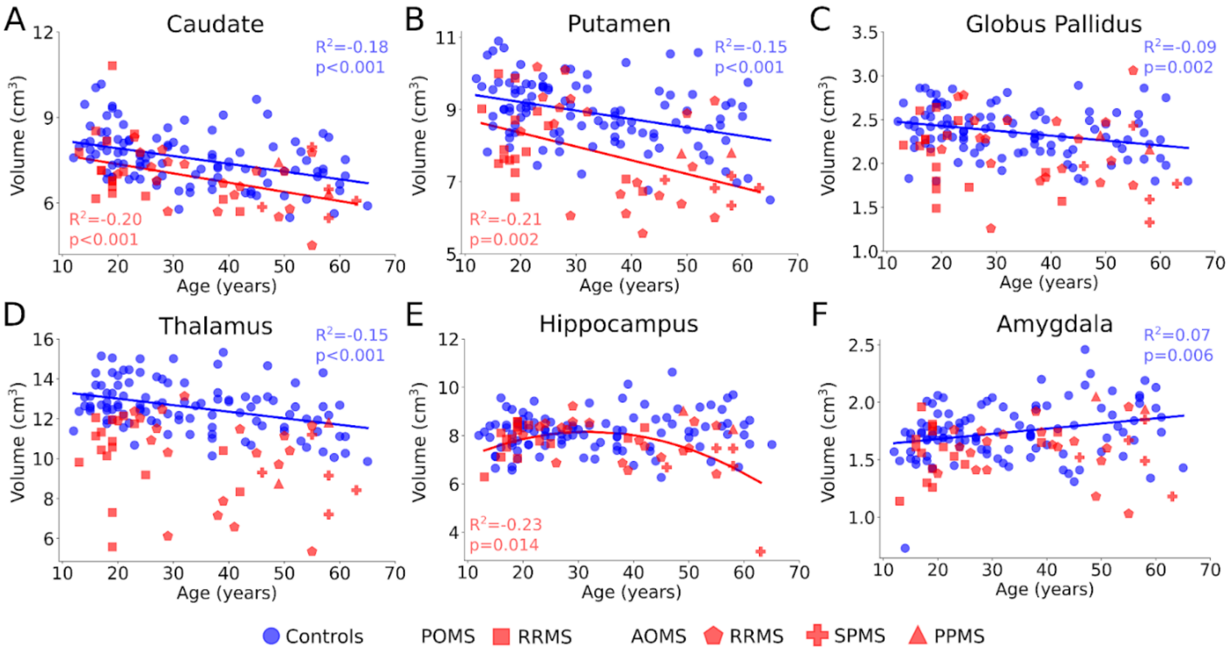

Supplemental Figure S3: Deep gray matter volumes (left+right) of (A) caudate, (B) putamen, (C) globus pallidus, (D) thalamus, (E) hippocampus and (F) amygdala versus age for controls (n=103, blue) and pediatric- and adult-onset MS (n=42, red). Most (4/6 regions; caudate, putamen, globus pallidus and thalamus) deep GM volumes in controls had negative linear age correlations; amygdala showed a positive correlation. MS participants showed lower offset negative correlations with age in the caudate and putamen over the entire age span, while the age relationship was lost for globus pallidus, thalamus, and amygdala, presumably due to marked volume reductions across all ages, although several participants were below controls over the full age range. The hippocampus volume had no age correlation in controls, but had a quadratic change in MS, which started to decline after 32 years of age. Further, the hippocampus did not show volume differences in MS in the < 35 year olds, but was reduced (albeit still within the control range) at older ages (i.e. the hippocampus in MS overlapped with controls in the children/adolescents/young adults, but was smaller in the older MS participants). This was unlike the other five deep GM regions where many MS participants had lower volumes over the entire age span.
