# Supplementary material for: High-resolution fluid-suppressed diffusion tractography of the fornix across the healthy lifespan and deviations in multiple sclerosis
Source: Imaging Neurosci (Camb). 2026 Mar 30;4:IMAG.a.1186. doi: 10.1162/IMAG.a.1186 (PMC13037659; doi:10.1162/IMAG.a.1186)
Supplement: Supplementary Table S1 [file IMAG.a.1186_Table_S1.pdf]

Supplemental Table S1: Fornix volume/diffusion metrics and total/regional left + right WM/GM volumes for males vs females in controls (M n=27, F n=76) and MS (M n=10, F n=32) separately (mean +/- standard deviation and percent changes between control and MS males/females; FDR corrected; *p<0.05; n.s. is non-significant).

	**Control**	**MS**	**Difference (%)**	
Fx Volume (cm^3)	M 5.8 +/- 0.9<br>F 5.0 +/- 1.1<br>p=0.002*	M 4.0 +/- 1.0<br>F 3.6 +/- 0.9<br>p=0.589	-31<br>-28	p<0.001*<br>p<0.001*
Fx FA	M 0.43 +/- 0.02<br>F 0.43 +/- 0.02<br>p=0.253	M 0.39 +/- 0.03<br>F 0.40 +/- 0.03<br>p=0.589	-9<br>-7	p<0.001*<br>p<0.001*
Fx MD ($x10^{-3}$ mm^2/s)	M 1.05 +/- 0.05<br>F 1.05 +/- 0.06<br>p=0.969	M 1.13 +/- 0.10<br>F 1.15 +/- 0.09<br>p=0.589	+8<br>+10	p=0.008*<br>p<0.001*
Fx AD ($x10^{-3}$ mm^2/s)	M 1.59 +/- 0.06<br>F 1.58 +/- 0.06<br>p=0.969	M 1.63 +/- 0.15<br>F 1.69 +/- 0.09<br>p=589	+3<br>+7	p=0.260<br>p<0.001*
Fx RD ($x10^{-3}$ mm^2/s)	M 0.78 +/- 0.05<br>F 0.79 +/- 0.06<br>p=0.969	M 0.87 +/- 0.09<br>F 0.89 +/- 0.09<br>p=0.589	+12<br>+13	p<0.001*<br>p<0.001*
CSF (cm^3)	M 212 +/- 46<br>F 160 +/- 40<br>p<0.001*	M 247 +/- 72<br>F 215 +/- 76<br>p=0.368	+17<br>+34	p=0.112<br>p<0.001*
Lateral Ventricles (cm^3)	M 13.6 +/- 7.6<br>F 10.5 +/- 6.0<br>p=0.041*	M 25.4 +/- 18.5<br>F 18.8 +/- 13.2<br>p=0.368	+87<br>+79	p=0.016*<br>p<0.001*
TBV (cm^3)	M 1383 +/- 87<br>F 1245 +/- 96<br>p<0.001*	M 1287 +/- 131<br>F 1150 +/- 126<br>p=0.024*	-7<br>-8	p=0.027*<br>p<0.001*
Total WM (cm^3)	M 592 +/- 48<br>F 530 +/- 53<br>p<0.001*	M 540 +/- 79<br>F 449 +/- 74<br>p=0.024*	-9<br>-15	p=0.036*<br>p<0.001*
Total GM (cm^3)	M 791 +/- 62<br>F 716 +/- 70<br>p<0.001*	M 747 +-/ 70<br>F 702 +/- 71<br>p=0.289	-6<br>-2	p=0.099<br>p=0.365

Cerebellum (cm^3)	M 154 +/- 13<br>F 141 +/- 13<br>p<0.001*	M 143 +/- 22<br>F 131 +/- 19<br>p=0.316	-7<br>-7	p=0.099<br>p=0.007*
Cerebellum WM (cm^3)	M 40 +/- 7<br>F 38 +/- 7<br>p=0.166	M 37 +/- 9<br>F 29 +/- 6<br>p=0.024*	-8<br>-23	p=0.285<br>p<0.001*
Cerebellum GM (cm^3)	M 114 +/- 13<br>F 103 +/- 12<br>p<0.001*	M 106 +/- 16<br>F 102 +/- 15<br>p=0.475	-7<br>-1	p=0.175<br>p=0.751
Caudate (cm^3)	M 7.7 +/- 0.9<br>F 7.5 +/- 1.0<br>p=0.483	M 7.2 +/- 1.6<br>F 6.8 +/- 0.9<br>p=0.475	-6<br>-9	p=0.236<br>p=0.002*
Putamen (cm^3)	M 9.3 +/- 0.8<br>F 8.7 +/- 0.9<br>p=0.005*	M 8.2 +/- 1.4<br>F 7.7 +/- 1.2<br>p=0.368	-12<br>-11	p=0.008*<br>p<0.001*
Globus Pallidus (cm^3)	M 2.4 +/- 0.3<br>F 2.3 +/- 0.3<br>p=0.119	M 2.2 +/- 0.6<br>F 2.1 +/- 0.4<br>p=0.475	-8<br>-9	p=0.112<br>p=0.001*
Thalamus (cm^3)	M 13.2 +/- 1.3<br>F 12.4 +/- 1.2<br>p=0.005*	M 10.7 +/- 2.1<br>F 9.8 +/- 2.0<br>p=0.368	-19<br>-21	p<0.001*<br>p<0.001*
Hippocampus (cm^3)	M 8.7 +/- 0.8<br>F 7.9 +/- 0.7<br>p<0.001*	M 8.0 +/- 0.8<br>F 7.5 +/- 1.1<br>p=0.368	-8<br>-5	p=0.038*<br>p=0.040*
Amygdala (cm^3)	M 1.9 +/- 0.2<br>F 1.7 +/- 0.2<br>p<0.001*	M 1.7 +/- 0.2<br>F 1.6 +/- 0.3<br>p=0.475	-11<br>-6	p=0.003*<br>p=0.083
