# Supplementary material for: High-resolution fluid-suppressed diffusion tractography of the fornix across the healthy lifespan and deviations in multiple sclerosis
Source: Imaging Neurosci (Camb). 2026 Mar 30;4:IMAG.a.1186. doi: 10.1162/IMAG.a.1186 (PMC13037659; doi:10.1162/IMAG.a.1186)
Supplement: Supplementary Table S2 [file IMAG.a.1186_Table_S2.pdf]

Supplemental Table S2: Pearson correlation coefficients, R, (top) and p-values (bottom) for MS fornix volume/diffusion metrics and total/regional left + right WM/GM volumes vs clinical/cognitive scores, FDR corrected; ***p<0.05**

	Fx vol	Fx FA	Fx MD	Fx AD	Fx RD	CSF	LV	TBV	WM	GM	Cerebellum	Cerebellum WM	Cerebellum GM	Caudate	Putamen	GP	Thalamus	Hippo	Amygdala
Time since MS onset (y)	-0.32<br>0.101	-0.26<br>0.220	0.14<br>0.557	0.01<br>0.985	0.21<br>0.342	**0.47**<br>**0.007***	**0.54**<br>**0.001***	**-0.52**<br>**0.003***	-0.30<br>0.100	**-0.64**<br>**<0.001***	**-0.40**<br>**0.022***	-0.23<br>0.235	**-0.41**<br>**0.017***	**-0.54**<br>**0.001***	**-0.58**<br>**0.001***	**-0.43**<br>**0.013***	**-0.49**<br>**0.004***	-0.34<br>0.052	-0.13<br>0.540
EDSS	-0.38<br>0.053	-0.18<br>0.463	-0.01<br>0.985	-0.15<br>0.557	0.08<br>0.860	**0.61**<br>**<0.001***	**0.52**<br>**0.003***	-0.35<br>0.052	-0.15<br>0.476	**-0.48**<br>**0.006***	**-0.42**<br>**0.016***	-0.29<br>0.122	**-0.41**<br>**0.017***	**-0.45**<br>**0.010***	**-0.51**<br>**0.003***	**-0.41**<br>**0.017***	**-0.37**<br>**0.036***	**-0.44**<br>**0.012***	0.03<br>0.912
TLV (cm^3)	**-0.50**<br>**0.010***	**-0.60**<br>**0.002***	**0.48**<br>**0.013***	0.24<br>0.261	**0.58**<br>**0.002***	**0.64**<br>**<0.001***	**0.39**<br>**0.022***	**-0.47**<br>**0.007***	**-0.56**<br>**0.001***	-0.24<br>0.207	**-0.49**<br>**0.004***	**-0.52**<br>**0.003***	**-0.40**<br>**0.021***	**-0.44**<br>**0.010***	**-0.50**<br>**0.004***	**-0.52**<br>**0.003***	**-0.85**<br>**<0.001***	-0.07<br>0.744	-0.01<br>0.960
Pediatric Fatigue (t-score)	-0.08<br>0.921	-0.01<br>0.985	0.11<br>0.876	0.12<br>0.875	0.10<br>0.88	-0.19<br>0.580	-0.21<br>0.555	-0.21<br>0.555	-0.06<br>0.899	-0.40<br>0.206	-0.16<br>0.657	-0.03<br>0.947	-0.19<br>0.580	-0.46<br>0.132	-0.37<br>0.256	-0.19<br>0.586	0.12<br>0.744	-0.35<br>0.278	**0.69**<br>**0.010***
MFIS (z-score)	-0.16<br>0.613	-0.18<br>0.557	-0.08<br>0.876	-0.18<br>0.557	-0.02<br>0.985	0.20<br>0.476	0.17<br>0.550	0.11<br>0.688	0.25<br>0.340	-0.06<br>0.838	0.10<br>0.714	0.22<br>0.422	0.03<br>0.911	0.04<br>0.899	-0.16<br>0.565	0.17<br>0.554	0.08<br>0.772	-0.06<br>0.853	0.20<br>0.476
BDI-II	-0.06<br>0.882	-0.01<br>0.985	-0.01<br>0.985	-0.01<br>0.985	-0.01<br>0.985	-0.09<br>0.658	0.01<br>0.948	0.11<br>0.580	0.18<br>0.400	0.01<br>0.947	0.07<br>0.757	0.12<br>0.580	0.03<br>0.900	-0.14<br>0.539	-0.02<br>0.947	0.13<br>0.558	0.12<br>0.580	0.01<br>0.947	0.17<br>0.417
SDMT (z-score)	**0.52**<br>**0.007***	0.24<br>0.252	-0.31<br>0.120	-0.20<br>0.344	-0.34<br>0.079	**-0.52**<br>**0.003***	-0.27<br>0.135	**0.62**<br>**<0.001***	**0.54**<br>**0.001***	**0.56**<br>**0.001***	**0.55**<br>**0.001***	**0.55**<br>**0.001***	**0.46**<br>**0.009***	**0.58**<br>**0.001***	**0.56**<br>**0.001***	**0.51**<br>**0.003***	**0.57**<br>**0.001***	**0.37**<br>**0.033***	0.15<br>0.485
BVMT-R (z-score)	0.27<br>0.208	0.25<br>0.252	-0.35<br>0.079	-0.26<br>0.228	-0.37<br>0.057	**-0.42**<br>**0.016***	-0.23<br>0.241	**0.50**<br>**0.004***	**0.43**<br>**0.013***	**0.46**<br>**0.009***	**0.63**<br>**<0.001***	**0.50**<br>**0.004***	**0.59**<br>**0.001***	**0.47**<br>**0.008***	0.38<br>0.234	**0.41**<br>**0.017***	**0.44**<br>**0.012***	**0.45**<br>**0.010***	0.22<br>0.268
T25FW (s)	**-0.56**<br>**0.036***	**-0.63**<br>**0.013***	**0.56**<br>**0.036***	0.29<br>0.344	**0.63**<br>**0.013***	**0.57**<br>**0.014***	0.25<br>0.400	**-0.60**<br>**0.010***	**-0.64**<br>**0.006***	-0.39<br>0.132	**-0.60**<br>**0.010***	**-0.52**<br>**0.028***	**-0.51**<br>**0.034***	-0.46<br>0.060	**-0.53**<br>**0.024***	**-0.59**<br>**0.011***	**-0.71**<br>**0.001***	-0.29<br>0.298	-0.07<br>0.843
9HPT (s)	**-0.57**<br>**0.034***	**-0.53**<br>**0.048***	0.45<br>0.099	0.21<br>0.557	**0.53**<br>**0.049***	**0.50**<br>**0.035***	0.20<br>0.496	**-0.52**<br>**0.026***	**-0.72**<br>**0.001***	-0.12<br>0.685	**-0.61**<br>**0.009***	**-0.60**<br>**0.010***	-0.46<br>0.057	-0.21<br>0.478	-0.40<br>0.120	-0.43<br>0.085	**-0.56**<br>**0.016***	-0.11<br>0.706	0.34<br>0.206
